# Lipotoxicity in Diabetic Cardiomyopathy: Molecular Basis and Emerging Therapeutic Targets

**DOI:** 10.3390/ijms27062740

**Published:** 2026-03-17

**Authors:** Yihua Han, Xinyi Chen, Oveena Fonseka, Wei Liu

**Affiliations:** Faculty of Biology, Medicine and Health, The University of Manchester, Manchester M13 9PT, UK

**Keywords:** lipotoxicity, heart failure, diabetic cardiomyopathy, cell metabolism, diabetes, lipid metabolism

## Abstract

Diabetic cardiomyopathy (DbCM) is an important contributor to heart failure (HF) in diabetes, occurring independently of other cardiovascular risk factors. Accumulating evidence demonstrates that cardiac lipotoxicity is a key driver of the onset and progression of DbCM and HF. Myocardial lipid homeostasis is coordinated by multiple transcriptional regulations, signaling pathway activation, and endoplasmic reticulum-mediated management involved in lipid metabolism. In DbCM, unbalanced fatty acid (FA) influx, handling, storage, and utilization initiates lipid overload, accumulation of toxic lipid intermediates (e.g., diacylglycerols and ceramides), and activation of maladaptive response. Notably, these lipid intermediates amplify reactive oxygen species (ROS) generation, which serves as a critical link between lipotoxic signaling and mitochondrial dysfunction by promoting electron leak, mitochondrial damage, and activation of inflammatory and cell-death pathways. These processes converge on adverse remodeling and contractile impairment, accelerating DbCM progression. This review integrates mechanistic and translational evidence linking dysregulated lipid handling to DbCM and discusses the potential therapeutic strategies that target lipid abnormalities.

## 1. Introduction

Diabetic cardiomyopathy (DbCM) is an increasingly recognized contributor to heart failure (HF) across the diabetic spectrum and occurs even in the absence of other cardiovascular risk factors. A defining feature of DbCM is impaired myocardial metabolic flexibility, in which chronic nutrient surplus and insulin resistance shift cardiomyocytes toward dominant fatty acid (FA) utilization while suppressing glucose use, creating a substrate imbalance that predisposes to lipotoxic injury. In the healthy heart, lipid homeostasis is maintained by coordinated control of FA uptake, oxidation, and storage through transcriptional and nutrient-sensing networks. These include the peroxisome proliferator-activated receptors (PPARs)-regulated oxidative programs, AMP-activated protein kinase (AMPK) signaling, and endoplasmic reticulum (ER)-Golgi pathways that couple lipid synthesis to stress adaptation via sterol regulatory element-binding proteins (SREBPs) and spliced X-box binding protein 1 (XBP1s)-linked proteostasis. In diabetes, these regulatory systems are disrupted and, hence, enable excessive FA delivery and uptake and promote the accumulation of bioactive lipid intermediates (e.g., diacylglycerols and ceramides) that activate various maladaptive signaling pathways. The chronic lipid overload further converges on inflammatory response, cell death, mitochondrial dysfunction, and elevated reactive oxygen species (ROS). This review synthesizes mechanistic and translational evidence linking dysregulated lipid handling and organelle stress to DbCM and provides current therapeutic strategies to mitigate its progression.

## 2. Epidemiology of Diabetes Mellitus

Diabetes mellitus (DM) is a heterogeneous disorder characterized by hyperglycemia, insulin deficiency, and/or insulin resistance. DM is one of the most prevalent chronic diseases worldwide, with increasing prevalence annually. According to the International Diabetes Federation statistics, 589 million adults aged 20–79 years were living with DM in 2024, and this number is estimated to increase to 853 million by 2050 [1]. Importantly, the International Diabetes Federation (IDF) projections indicate that growth will be greatest in developing regions (e.g., Africa +142%; Middle East and North Africa +95% by 2050) and comparatively smaller in industrialized regions (e.g., Europe +10%; North America and the Caribbean +21%) [1]. In the meantime, the incidence of cardiovascular diseases (CVDs) is projected to continue rising, which can be attributed to the prevalence of obesity and diabetes continuing to increase at an alarming rate. The number of CVD deaths increased globally from 13.1 million in 1990 to 19.2 million in 2023 [2]. DM markedly increases CVD risks as a complication and may develop to the lethal stage, which is the leading cause of mortality and disability among patients with DM [3]. In the WHO Multinational Study of Vascular Disease in Diabetes, a multinational cohort of patients with DM, cardiovascular disease emerged as the leading underlying cause of death, accounting for 44% of deaths in individuals with type 1 diabetes and 52% of deaths in those with type 2 diabetes mellitus (T2DM) [4]. The risk of CVD increases proportionally with fasting blood sugar levels, even before blood glucose reaches diabetic thresholds [5].

## 3. Vascular Complications of DM

### 3.1. Microvascular Complications

Diabetic retinopathy, nephropathy, and neuropathy classically reflect small-vessel injury driven by chronic hyperglycemia and hemodynamic/metabolic stressors, driving a cascade of advanced glycation end products (AGEs) and receptor of AGEs signaling, polyol and hexosamine pathway flux, protein kinase C (PKC) activation, mitochondrial ROS, ER stress, endothelial dysfunction, and low-grade inflammation that culminate in capillary basement-membrane thickening and tissue ischemia [6,7]. Clinically, these complications manifest as vision loss, kidney dysfunction, and peripheral/autonomic neuropathy [6,8]. Beyond organ-specific morbidity, generalized microvascular rarefaction and endothelial dysfunction promote myocardial fibrosis and impaired relaxation, linking microvascular disease to diastolic dysfunction and HF in DM [9].

### 3.2. Macrovascular Complications

Macrovascular complications are driven by the interplay of chronic hyperglycemia; insulin resistance; and atherogenic dyslipidemia, including elevated triglycerides (TGs), lower high-density lipoprotein, higher small dense low-density lipoprotein (LDL), endothelial dysfunction, low-grade inflammation, oxidative stress, and prothrombotic signaling, which accelerate plaque formation and impair vasomotor tone [10,11]. Clinically, this translates to a higher and earlier burden of coronary artery disease, multivessel involvement, and greater risk of myocardial infarction [12]; cerebrovascular disease with ischemic stroke and cerebral hemorrhage [13]; and peripheral artery disease characterized by intermittent claudication [14].

## 4. Diabetic Cardiomyopathy (DbCM) and Heart Failure (HF)

It is important to note that even in patients with prediabetes, the risk of HF is increased and associated with poor prognosis [15]. Indeed, it has long been recognized that individuals with T2DM have an elevated risk for developing HF irrespective of coronary heart disease and/or hypertension [16].

DbCM represents a distinct clinical entity, first proposed by Lundbaek in 1954 [17], of the existence of a specific diabetic heart muscle disease without involvement of coronary artery diseases or hypertension. In 1972, Rubler et al. [18] provided the first clinical characterization of DbCM to report post-mortem data from four T2DM patients who died of HF without evidence of cardiovascular risk factors other than diabetes. DbCM was first professionally defined in 2013 by a collaborative effort involving the American College of Cardiology Foundation, the American Heart Association [19], the European Society of Cardiology, and the European Association for the Study of Diabetes [20]. The resulting definition specifies a clinical condition of ventricular dysfunction in patients with DM that manifests independently of coronary atherosclerosis and hypertension.

According to the Framingham Heart Study, T2DM is associated with an increased 2.4- and 5-fold higher risk of HF and cardiovascular mortality in men and women, respectively, compared with age-matched individuals after adjustment for common CVD risk factors [21]. Similar results were obtained from another observational cohort of more than 8000 patients followed for up to 6 years; the risk of new-onset HF was 2.5-fold higher in patients with T2DM than their non-diabetic counterparts [22]. Meanwhile, in T1DM, each 1% increase in glycated hemoglobin was associated with approximately a 30% higher risk of HF in a large Swedish national cohort of 20,985 participants [23]. Similarly, in the United Kingdom prospective diabetes study cohort of patients with T2DM, each 1% higher updated mean glycated hemoglobin was associated with roughly a 15–20% higher risk of HF after adjustment for age, sex, ethnicity, blood pressure, lipids, smoking, and albuminuria [24], supporting the concept that graded increases in glycemia are an important promoter of HF in diabetic patients.

HF is classified by left ventricular ejection fraction (LVEF) into distinct phenotypes: HFpEF (heart failure with preserved ejection fraction, LVEF ≥ 50%), defined by preserved systolic function but impaired diastolic reserve with elevated filling pressures, commonly driven by myocardial stiffening from interstitial fibrosis cross-linking and cardiomyocyte passive stiffening (e.g., titin hypophosphorylation) on a background of microvascular dysfunction and low-grade inflammation [25]. HFrEF (heart failure with reduced ejection fraction, LVEF ≤ 40%) is characterized by systolic dysfunction with eccentric remodeling and LV dilation following cumulative cardiomyocyte injury and more maladaptive extracellular matrix remodeling [26]. HFmrEF (heart failure with mildly reduced ejection fraction, LVEF 41–49%) is an intermediate phenotype with overlapping features and heart failure with improved ejection fraction, describing patients with prior HFrEF (LVEF ≤ 40%) whose LVEF improves to >40% with therapy, typically accompanied by reverse remodeling (reduced LV volumes) [27]. In T2DM, HF is highly prevalent and HFpEF appears to be the dominant clinical phenotype in contemporary epidemiologic data [28]. Given that HFpEF in T2DM is typically enriched for obesity/metabolic syndrome, lipid oversupply and maladaptive lipid partitioning are likely to play a more proximal role in driving cardiomyocyte lipotoxic signaling. By contrast, HFrEF is more often initiated by ischemic injury, yet lipotoxic lipid intermediates—particularly sphingolipids/ceramides—are also observed in HFrEF (including advanced disease) and may contribute to ongoing mitochondrial dysfunction, oxidative stress, and adverse remodeling, with the relative contribution likely varying by HF etiology and stage [29].

## 5. Lipid-Involved Mechanisms Underlying DbCM

The pathophysiology of DbCM is multifactorial, involving ER dysfunction, mitochondrial dysfunction, and myocardial lipid overload [30,31]. Chronic hyperglycemia and other metabolic insults impair cardiomyocyte metabolic flexibility and promote oxidative stress, leading to cardiac lipotoxicity and cardiac pathological remodeling [32].

### 5.1. Physiological Lipid Metabolism in Cardiomyocytes

Due to the high energy demand, the healthy heart is metabolically flexible and can readily shift between different energy substrates, which include FAs accounting for 40–60% ATP production, carbohydrates (i.e., glucose), ketone bodies, and amino acids to maintain ATP production [33]. These must be acquired continuously from the blood due to the heart’s low ability to store these energy substrates intracellularly [34]. The majority of the oxygen consumed by the heart is utilized for mitochondrial oxidative phosphorylation (OXPHOS) via the electron transport chain (ETC), whereas the synthesis of ATP derived from glycolysis does not require oxygen.

FAs are delivered to the cardiomyocytes either as non-esterified FAs bound to albumin in blood or as liberated FAs hydrolyzed from TGs contained in chylomicrons and very low-density lipoproteins. Cardiomyocytes can also internalize LDL particles via the LDL receptor (LDLR), providing a source of cholesterol and cholesteryl esters that can be hydrolyzed intracellularly to liberate fatty acids. The FAs are primarily transported across the sarcolemmal membrane via protein-mediated transport or passive diffusion [35]. One of the major protein transporters that has been shown to mediate ~50% of cardiac FA uptake is cluster of differentiation 36 (CD36). FA transport proteins (FATPs) at the membrane for uptake/activation, including FATP1 and FATP6, and the heart-type FA binding protein (FABP3) and FABP4, function as an intracellular chaperone to transport FAs from the cell membrane to mitochondria and other organelles [36].

FAs are first activated by being esterified to coenzyme A (CoA) via fatty acyl-CoA synthetases on the outer mitochondrial membrane or other cellular membranes, forming long-chain acyl-CoA. These long-chain acyl-CoAs are then converted into long-chain acylcarnitines by carnitine palmitoyltransferase 1 (CPT1), which has tissue-specific isoforms like CPT1b (muscle/heart isoform), located on the outer mitochondrial membrane [37]. The long-chain acylcarnitines are transported across the inner mitochondrial membrane by the carnitine–acylcarnitine translocase and then converted back into long-chain acyl-CoAs by CPT2, which is located on the matrix-facing side of the inner mitochondrial membrane [38]. This fatty acyl-CoA is catabolized through a helical sequence of reactions known as β-oxidation, yielding multiple units of acetyl-CoA that condense with oxaloacetate, forming citrate to enter the tricarboxylic acid (TCA) cycle, along with the reducing equivalents: NADH_2_ (commonly referred to as NADH, nicotinamide adenine dinucleotide) and FADH_2_ (flavin adenine dinucleotide). NADH_2_ and FADH_2_ are subsequently oxidized by the respiratory chain (Complex I and electron-transfer flavoprotein/ubiquinone entry, respectively), delivering electrons to ubiquinone and ultimately to O_2_ to form H_2_O. Simultaneously, the coupled proton pumping across the inner mitochondrial membrane establishes the proton gradient that drives ATP synthesis via oxidative phosphorylation [39].

**Figure 1:**
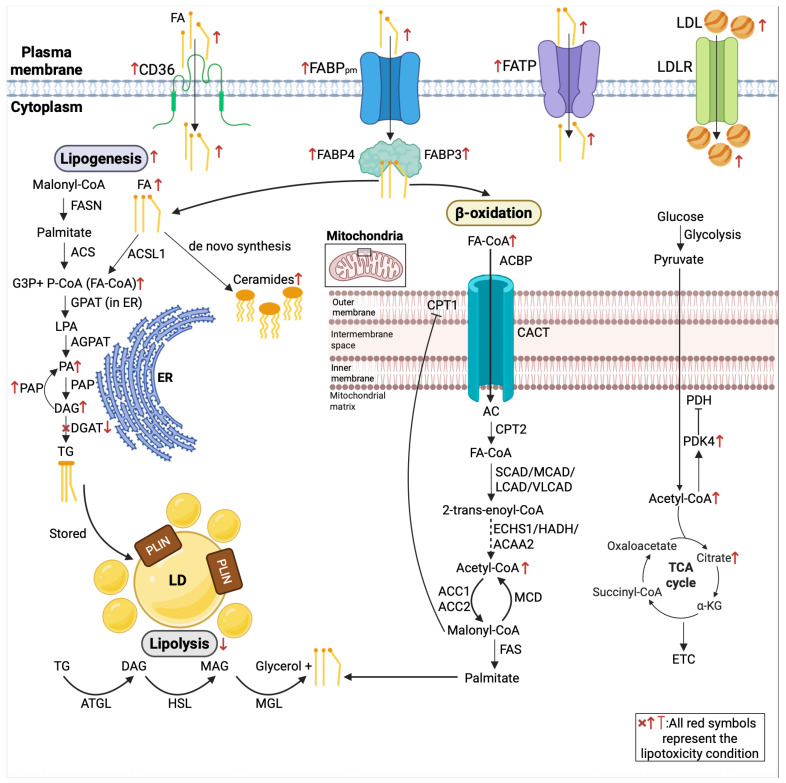
A schematic overview of myocardial lipid uptake, handling, storage, and oxidation in cardiomyocytes and the remodeling under diabetic/lipotoxic conditions. Maintaining a balance between fatty-acid uptake, intracellular trafficking, esterification/storage, lipolysis, and mitochondrial β-oxidation is essential for efficient ATP generation and contractile function; disruption of this balance promotes lipid overload and maladaptive metabolic remodeling. Solid arrows indicate direct processes; dotted arrows denote indirect/condensed connections with omitted intermediates. Red symbols indicate changes in lipotoxic/diabetic states. ACAA2, acetyl-CoA acyltransferase 2; ACBP, acyl-CoA-binding protein; ACC1/ACC2, acetyl-CoA carboxylase 1/2; AGPAT, 1-acylglycerol-3-phosphate acyltransferase; ACSL1, long-chain acyl-CoA synthetase 1; ATGL, adipose triglyceride lipase; CACT, carnitine–acylcarnitine translocase; CD36, cluster of differentiation 36; CPT1, carnitine palmitoyltransferase 1; CPT2, carnitine palmitoyltransferase 2; DAG, diacylglycerol; DGAT, diacylglycerol acyltransferase; ECHS1, enoyl-CoA hydratase short chain 1; ER, endoplasmic reticulum; ETC, electron transport chain; FA, fatty acid; FA-CoA, fatty acyl-CoA; FABPpm, plasma membrane fatty acid-binding protein; FABP3/4, fatty acid-binding protein 3/4; FASN, fatty acid synthase; FATP, fatty acid transport protein; G3P, glycerol-3-phosphate; GPAT, glycerol-3-phosphate acyltransferase; HADH, 3-hydroxyacyl-CoA dehydrogenase; HSL, hormone-sensitive lipase; LD, lipid droplet; LDL, low-density lipoprotein; LDLR, LDL receptor; LPA, lysophosphatidic acid; MAG, monoacylglycerol; SCAD/MCAD/LCAD/VLCAD, short-chain/medium-chain/long-chain/very long chain acyl-CoA dehydrogenase; MCD, malonyl-CoA decarboxylase; MGL, monoacylglycerol lipase; PA, phosphatidic acid; PAP, phosphatidic acid phosphatase; PDH, pyruvate dehydrogenase; PDK4, pyruvate dehydrogenase kinase 4; PLIN, perilipin; TCA, tricarboxylic acid cycle; TG, triacylglyceride.

Prior to β-oxidation, free FAs can be stored in the form of TGs as lipid droplets (LDs) coated with surface proteins perilipin 1–5, which is another key source of free FAs in cardiomyocytes [40]. The process in which TGs are synthesized is also known as lipogenesis. Initially in lipogenesis, glycerol is converted to glycerol-3-phosphate by glycerol kinase [41]. Then, an acyl-CoA is added to the first carbon of glycerol-3-phosphate to form lysophosphatidic acid, catalyzed by glycerol-3-phosphate acyltransferase (GPAT) [42]. This is followed by the addition of acyl-CoA onto the second carbon, catalyzed by acylglycerolphosphate acyltransferase to form phosphatidate [43]. After that, phosphatidic acid phosphatase catalyzes the conversion of phosphatidate into diacylglycerol (DAG). Then, diacylglycerol acyltransferase (DGAT) drives the formation of TGs by the addition of an acyl-CoA molecule [44]. These TGs are hydrolyzed by the process of lipolysis, which is elevated under low levels of insulin and glucose. TGs are initially hydrolyzed by adipose triglyceride lipase (ATGL) with TGs to form DAGs and a free FA [45]. DAGs are converted to monoacylglycerols (MAGs) plus an FA by hormone-sensitive lipase (HSL), which is followed by digestion into glycerol and FA by monoglyceride lipase (MGL). HSL is also able to catalyze the hydrolysis of TGs and MAGs in the absence of ATGL and MGL, respectively [46] (Figure 1).

### 5.2. Key Molecular Basis Regulating Lipid Homeostasis

PPARs are a subfamily of nuclear receptor transcription factors that regulate gene expression in response to endogenous lipid ligands, including three main isoforms, PPARα, PPARβ/δ, and PPARγ. Several canonical PPARα target genes have been identified in the heart, forming a coordinated regulatory network that enables efficient lipid utilization. These include *CD36* and *FATPs*; *acyl-CoA synthetase long-chain family member 1* (*ACSL1*), which catalyzes lipid activation; and *CPT1*. In addition, PPARα enhances the transcription of mitochondrial β-oxidation enzymes, including the chain-length-specific acyl-CoA dehydrogenases SCAD, MCAD, LCAD, and VLCD (short-chain/medium-chain/long-chain/very long chain acyl-CoA dehydrogenase) [47], which catalyze the initial dehydrogenation step in β-oxidation for different lengths of FAs, thereby sustaining FA catabolism and ATP production in cardiomyocytes. Activation of PPARβ/δ in cardiomyocytes induces a broad transcriptional program that supports mitochondrial and peroxisomal fatty acid oxidation (FAO), including upregulating the expression level of CPT1, VLCAD, LCAD, and malonyl-CoA decarboxylase (MCD). In diabetic failing hearts, the coactivator PPARγ coactivator-1α (PGC-1α) is reduced, leading to mitochondrial dysfunction associated with lipid overload [48].

AMPK is a highly conserved serine/threonine kinase that serves as a master regulator of cellular energy homeostasis. As regulating lipid metabolism is the first known function of AMPK, AMPK orchestrates multiple changes to reduce energy storage and enhance energy production [49]. These include the suppression of de novo FA synthesis, cholesterol biosynthesis, and TGs accumulation, as well as the stimulation of FA uptake and mitochondrial FAO. Once activated, AMPK coordinates a metabolic shift by inhibiting key lipid biosynthetic enzymes (e.g., ACC1, GPAT, and 3-hydroxy-3-methylglutaryl-CoA reductase) and by restraining lipogenic transcriptional programs (e.g., SREBP1c) [50]. Conversely, diabetic nutrient surplus is frequently accompanied by impaired AMPK activity, which removes this inhibitory “brake” on lipid anabolism and perturbs malonyl-CoA/CPT1 control, thereby favoring lipid accumulation and lipotoxic remodeling [35,50]. Mechanistically, AMPK promotes mitochondrial FA oxidation via the ACC2–malonyl-CoA–CPT1 axis and increases FA uptake by driving CD36 translocation to the sarcolemma through liver kinase B1–AMPK signaling and phosphorylation of Rab GTPase-activating proteins, enabling Rab-dependent vesicular trafficking rather than a defined AMPK-specific transcriptional pathway for *CD36* [51].

The ER has shown to be crucial for regulating lipid homeostasis in cells, because most factors related to lipid metabolism are located on the ER, playing an important role in lipid homeostasis [52]. Upon acute stress, the XBP1s is increased, serving as a potent transcription factor upregulating the expression of ER chaperones like 78-kDa glucose-regulated protein and calnexin that aid protein folding or enhance misfolded protein degradation [53,54]. In the heart, XBP1s has emerged as a cardiomyocyte lipid rheostat. In human diabetic hearts, the reduction of XBP1s is associated with HFpEF. Furthermore, in mouse HFpEF models, it limits steatosis by promoting factor forkhead box protein O1 (FoxO1) proteasomal degradation through upregulating the expression of the E3 ubiquitin ligase STUB1, which otherwise drives lipid accumulation in cardiomyocytes [55]. Recent work further shows that XBP1s-mediated ER function mitigates cardiac lipotoxicity by promoting SEC23A-dependent ATGL trafficking and stabilization, thereby limiting LD accumulation in HFpEF models [54].

The SREBPs are a family of ER membrane-bound transcription factors that serve as master regulators of lipid metabolism. The three major isoforms of SREBPs family are encoded by two genes. SREBF1 gives rise to SREBP1a and SREBP1c through alternative promoter usage, whereas SREBF2 encodes SREBP2. While they are structurally similar, the isoforms differ in their target gene specificity and transcriptional potency. SREBP1a and SREBP2 have strong transactivation potential to the regulation of cholesterol biosynthetic pathways, notably HMG-CoA reductase and the LDLR [56]. SREBP1c, by contrast, preferentially activates genes involved in de novo lipogenesis, including fatty acid synthase (FASN), ACC, and stearoyl-CoA desaturase-1 [57]. ER-resident SREBP traffics to the Golgi, initiating the proteolytic maturation and its activation process [58]. In patients with metabolic syndrome, ventricular biopsies revealed significantly elevated SREBP1c mRNA and protein levels compared to non-MS controls. Histological analyses demonstrated prominent intracellular LDs and intense SREBP1c immunostaining, particularly in vacuolated cardiomyocytes [59]. Consistently, the animal diabetic model also shows increased activation of SREBP1c and its nuclear transactivation of lipogenic targets (e.g., *Acc*, *Fasn*, *Scd1*, and *Dgat2*), leading to myocardial lipid accumulation, impaired energetics, and contractile dysfunction [60]. In particular, SREBP1c expression correlated positively with cardiac lipid amount but negatively with cardiac contraction, implicating SREBP1c in the pathogenesis of DbCM [59,60].

## 6. Lipotoxicity in DbCM

**Figure 2:**
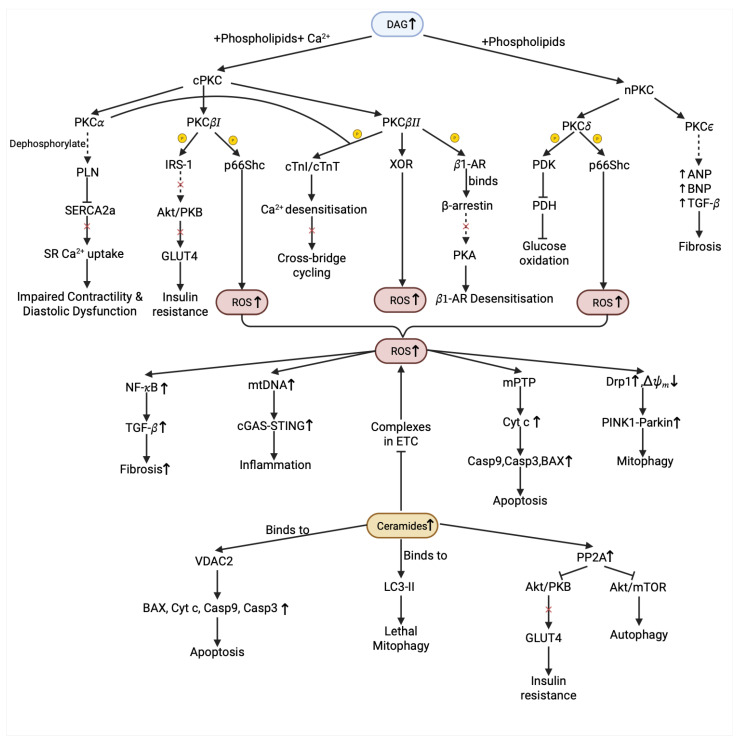
Lipotoxicity-driven signaling networks in diabetic cardiomyopathy. Under lipotoxic conditions, accumulation of the lipid intermediates diacylglycerol and ceramides engages distinct but interconnected signaling modules. Both DAG- and ceramide-initiated signaling amplify reactive oxygen species, which further propagate downstream programs, thereby reinforcing DbCM progression. Solid arrows indicate direct processes; dotted arrows denote indirect or condensed connections with omitted intermediates. Upward and downward arrows indicate increased and decreased activity, expression, or levels, respectively; red X marks indicate inhibition or impairment; and yellow circles labeled P indicate phosphorylation. Akt/PKB, protein kinase B; ANP, atrial natriuretic peptide; BAX, Bcl-2-associated X protein; β_1_-AR, β_1_-adrenergic receptor; BNP, B-type natriuretic peptide; Casp3, cysteinyl aspartate-specific protease-3; Casp9, cysteine-aspartic protease 9; cGAS, cyclic GMP–AMP synthase; cPKC, conventional protein kinase C; cTnI, cardiac troponin I; cTnT, cardiac troponin T; Cyt c, cytochrome c; DAG, diacylglycerol; Drp1, dynamin-related protein 1; ETC, electron transport chain; GLUT4, glucose transporter 4; IRS-1, insulin receptor substrate 1; LC3-II, microtubule-associated protein 1 light chain 3-II; mPTP, mitochondrial permeability transition pore; mtDNA, mitochondrial DNA; mTOR, mechanistic target of rapamycin signaling; NF-κB, nuclear factor-κB; nPKC, novel protein kinase C; P, phosphorylation; PDK, pyruvate dehydrogenase kinase; PDH, pyruvate dehydrogenase; PINK1, PTEN-induced kinase 1; PKA, protein kinase A; PKCα/βI/βII/δ/ε, protein kinase C alpha/beta I/beta II/delta/epsilon; PLN, phospholamban; PP2A, protein phosphatase 2A; p66Shc, 66 kDa Src homology 2 domain-containing protein; ROS, reactive oxygen species; SERCA2a, sarco/endoplasmic reticulum Ca^2+^-ATPase 2a; SR, sarcoplasmic reticulum; STING, stimulator of interferon genes; TGF-β, transforming growth factor-β; VDAC2, voltage-dependent anion channel 2; XOR, xanthine oxidoreductase; Δ𝜓𝑚, mitochondrial membrane potential.

In this review, cardiac lipotoxicity refers to lipid oversupply and dysregulated lipid handling in cardiomyocytes that promote the accumulation of bioactive lipid intermediates and trigger cellular dysfunction, inflammation, and cell death. While additional mediators (e.g., phosphatidic acid, eicosanoids, and endocannabinoids) may contribute, we focus on three representative lipotoxic pathways in DbCM: DAG signaling, ROS-mediated stress, and ceramide-driven injury. Importantly, lipotoxicity is not only a pathological consequence of lipid overload in non-adipose tissues but also a key driver of myocardial injury in many cardiac abnormalities. In the diabetic heart, increased FA delivery and uptake promote a shift toward predominant FA oxidation with reciprocal suppression of glucose utilization, consequently contributing to various intracellular toxic effects and cell death and accelerating the onset and development of DbCM and HF [61]. Substrate imbalance favors intracellular undue storage of TGs and, more importantly, accumulation of bioactive lipid intermediates. Human evidence from heterotopic heart transplantation shows that exposure of a non-diabetic donor heart to DM results in marked myocardial lipid accumulation [62]. The abnormal lipid accumulation in cells disrupts the lipid composition in cell membranes, causing the destabilization of ion channels [63]. Excessive lipids also impair mitochondrial function, aggravate oxidative stress, and disrupt cellular homeostasis [61]. Furthermore, lipotoxic stress can promote cardiac inflammation, apoptosis and pathological remodeling [64], which collectively intensify cellular damage and facilitate the structural and functional deterioration of the heart (Figure 2).

### 6.1. Unbalanced Lipid Metabolism

Studies have demonstrated that the genes involved in lipid metabolism are broadly dysregulated in the diabetic heart. Increased myocardial FA uptake represents an initiating event in cardiac lipotoxicity, with rodent studies showing that enhanced FA influx promotes the accumulation of toxic lipid metabolites and leads to lipotoxic cardiomyopathy [65,66,67]. CD36 has been shown to drive excessive lipid influx under conditions of metabolic disease [51]. Human genetic studies also have demonstrated that *CD36* expression is upregulated in obesity and T2DM to enhance cardiac FA uptake, whereas *CD36* mutations led to markedly reduced myocardial FA uptake [68,69]. In obesity and diabetes, increased CD36 expression and translocation to the sarcolemma markedly enhance FA uptake, as sarcolemma localization directly determines the rate of FA influx into cardiomyocytes [70]. In addition, this persistent CD36 translocation sustains excessive lipid entry, thereby promoting LD accumulation and lipotoxic stress, and subsequent cardiac dysfunction [71,72] (Figure 1).

Downstream of membrane entry, diabetes remodels intracellular FA trafficking and utilization. The FABP3 is increased in streptozotocin (STZ)-diabetic T1DM mouse hearts together with a shift toward FA catabolic programs, yet myocardial ATP is reduced in parallel with ultrastructural mitochondrial injury. This greater FA delivery to oxidation outstrips damaged mitochondrial capacity and lowers energetic efficiency [73]. In ambulatory chronic HF, circulating FABP3 and adipose tissue-specific FABP4 are higher in patients with T2DM, and in emerging research, they are potential biomarkers to independently predict all-cause and cardiovascular mortality in the T2DM subgroup [74]. Serum FABP4 is also elevated in HFpEF with worse clinical outcome and is associated with cardiac remodeling and dysfunction in a prospective observational cohort study [75]. In well-controlled T2DM patients without overt structural heart disease, serum FABP4 is also higher than in controls and is positively associated with myocardial neutral lipid [75]. Overall, the available clinical data are most consistent with circulating FABP4 serving as a consequence of cardiometabolic stress, whereas a direct endocrine or paracrine causal role in myocardial injury remains unproven in vivo. Mechanistically, adding recombinant FABP4 to HL-1 cardiomyocytes increases intracellular lipid accumulation and induces insulin-signaling defects with reduced insulin-stimulated AKT/AS160 signaling-impaired GLUT4 translocation and glucose uptake [76] (Figure 1). However, these findings derive from an acute in vitro exposure model, and whether circulating FABP4 causally drives analogous cardiomyocyte effects in vivo remains uncertain.

On the other hand, transcriptional programs that favor de novo lipogenesis can be activated in metabolically stressed myocardium. P21-activated kinase 3 (PAK3) is elevated in obese human myocardium and mice. PAK3 activation promotes nuclear SREBP1c through mechanistic target of rapamycin (mTOR)/ribosomal protein S6 kinase 1 (S6K1) signaling [77], driving an “abnormal lipid gene profile” with upregulation of FA uptake and lipogenesis genes (e.g., *Cd36*, *Fabp3*, *Acsl1*, *Acaca*, *Acacb*, and *Dgat2*) alongside suppression of FAO genes (e.g., *Cpt1b*, *Cpt2*, *Acadl*, and *Acadm*), thereby increasing myocardial toxic lipid accumulation and oxidative stress [59]. Beyond increased FA influx and biosynthesis, lipotoxic remodeling also involves impaired intracellular lipid clearance. Metabolic HFpEF is associated with suppression of the XBP1s–ER degradation-enhancing alpha-Mannosidase-like protein 2 axis, which promotes LD accumulation by disrupting SEC23A-dependent ATGL trafficking, accelerating ATGL degradation, and increasing myocardial TGs and DAG content and oxidative stress [53].

### 6.2. Lipotoxic Intermediates

In cardiomyocytes, a small but rapidly turning-over TG pool buffers the transient FA supply and demand; when FA delivery and oxidation are balanced, bioactive intermediates remain low and downstream signaling is quiescent [78]. In T2DM, FA influx exceeds mitochondrial FAO due to increased CD36 activity, diverting carbon into TGs, DAGs, and ceramides through serine palmitoyl transferase (SPT) of de novo sphingolipid, which leads to lipotoxicity [79]. Because DAG is the direct substrate for TGs synthesis, the terminal esterification of DAG to TG is catalyzed by DGAT, acting as a buffering “sink” for excess acyl-CoA. According to consistent observation in adult mouse hearts, combined attenuation of DGAT1/2 activity reduced TG turnover and blunted high-fat-diet-induced myocardial lipid accumulation without overt baseline functional deterioration [80]. Conversely, cardiomyocyte-restricted loss of DGAT1 in mice caused accumulation of DAG and ceramides with contractile impairment [81]. Excess DAG activates novel/conventional PKC group of enzymes, which phosphorylate proteins at serine and threonine residues [82]. The major PKC isoform expressed in cardiomyocytes is PKCα, which modulates contractility through directly phosphorylating protein phosphatase inhibitor-1, thereby disinhibiting protein phosphatase 1 activity, which in turn dephosphorylates phospholamban (PLN). Dephosphorylated PLN is a more potent inhibitor of sarco/endoplasmic reticulum Ca^2+^ ATPase 2a, ultimately reducing sarcoplasmic reticulum Ca^2+^ uptake and contractility [83]. PKCα also directly phosphorylates key myofilament proteins like cardiac Troponin I to reduce the myofilament’s sensitivity to Ca^2+^, which decreases maximal force generation and ultimately depresses overall cardiac contractility. Increased expression and activity of PKCα are strongly associated with HF, especially in the diastolic period and maladaptive ventricular remodeling, including promoting hypertrophy and fibrosis via galectin-3 and accumulation of collagen [84].

In addition, circulating ceramides are largely produced by metabolically active organs from the liver, where saturated FA oversupply drives de novo sphingolipid synthesis and subsequent export in lipoproteins. In clinical cohorts, panels of plasma ceramide species and composite scores (e.g., Cardiovascular Event Risk Test 1) therefore serve as systemic readouts of dysregulated lipid handling and have been proposed as potential biomarkers for cardiometabolic risk and stratification in diabetic heart-failure events [85]. Clinical evidence supports the pathogenic relevance of these mechanisms: a meta-analysis of three prospective clinical studies in Finland, Switzerland, and Norway that followed patients with CVD found that ratios of long-chain ceramide species (C18:1/C16:0 and C18:1/C18:0) were more strongly correlated with negative cardiovascular outcomes than ratios of very long chain ceramide species (C18:1/C24:0) [86]. Another supporting study demonstrated that the inhibition of SPT with myriocin, an enzyme involved in ceramide biosynthesis, shifted fuel use toward glucose, preserved systolic function and increased survival rates [87]. Mechanistically, ceramides act as bioactive signals of lipid overload that reprogram cardiomyocyte metabolism by concurrently impairing mitochondrial energetics and insulin signal transduction. In the mitochondria, ceramide accumulation is associated with reduced OXPHOS efficiency and increased oxidative stress, consistent with disruption of the ETC function. The electron entry through Complex I/II and transfer to Complex III require coenzyme Q (CoQ), and the elevation of mitochondrial ceramides has been linked to CoQ depletion and loss of respiratory chain components, with increasing electron leak, thereby elevating mitochondrial ROS production [88]. In cardiomyocytes, lipid-driven increases in very long chain ceramides via ceramide synthase 2 have been shown in a mouse model of DbCM to provoke mitochondrial dysfunction, oxidative stress, and mitophagy alongside insulin resistance [89]. Further study also illustrates that ceramide binding to voltage-dependent anion channel 2 facilitates pro-apoptotic Bax handling and promotes mitochondrial apoptosis [90]. In parallel, ceramide-induced insulin resistance via inhibition of AKT (aka PKB) signaling impairs myocardial glucose uptake and reinforces a maladaptive reliance on FA metabolism that sustains lipotoxic stress [91]. In cancer cells, C18-ceramides can directly engage the autophagy machinery by binding microtubule-associated protein 1A/1B-light chain 3B (LC3B-II) at mitochondrial membranes, thereby recruiting autophagolysosomes to mitochondria and driving lethal mitophagy in a dynamin-related protein 1 (DRP1)-dependent context [92]. Complementing this, a newly identified protein, p17/PERMIT, mediates ER–mitochondria trafficking that delivers ceramide synthase 1 from the ER to the outer mitochondrial membrane, spatially localizing ceramide biogenesis to mitochondria and linking ceramide production to mitochondrial protein import and stress [93].

### 6.3. Mitochondrial Dysfunction

It is established that higher FA uptake leads to greater β-oxidation. Such high oxidation levels can ultimately exceed the capacity of the TCA cycle, causing an accumulation of toxic molecules such as acylcarnitine as a result of partial β-oxidation [94]. Furthermore, in the hearts of animals with diabetes, the expression of MCD, which catalyzes the degradation of malonyl-CoA, is upregulated. As a result, the inhibition of CPT1 by malonyl-CoA is attenuated, leading to elevated fatty acyl-CoA transport into mitochondria [95]. Sakamoto et al. [96] report that STZ-induced diabetic mice with high levels of FA oxidation displayed increased expression of MCD. Furthermore, diabetes increases the expression of proton transporters known as uncoupling proteins (UCPs), which can reduce the proton gradient of the inner mitochondrial membrane. In early stages, for instance, UCP3 is upregulated in cardiomyocytes of rats with STZ-induced diabetes [97], which may serve as a compensatory mechanism to limit mitochondrial ROS generation by reducing electron leak; however, sustained or excessive uncoupling decreases energetic efficiency, leading to reduced ATP production and partial oxidation [35].

Diabetic lipid overload drives the accumulation of toxic lipid intermediates and perturbs cardiomyocyte protein-quality-control pathways, including ubiquitin–proteasome-mediated A-kinase-anchoring protein 1 degradation and stress-linked optic atrophy 1 proteolysis, thereby compromising mitochondrial structure and bioenergetics [98,99]. Ceramides can incorporate into mitochondrial membranes and perturb cardiolipin-rich domains, impairing ETC efficiency and increasing electron leak and ROS generation [91]. In parallel, DAG-driven activation of PKCδ promotes its mitochondrial targeting and phosphorylation of DRP1, driving mitochondrial fission or fragmentation and amplifying mitochondrial ROS, thereby worsening Ca^2+^-handling stress and weakening mitochondrial quality control [100]. This chronic fragmented state degrades cristae integrity and depresses ATP generation, ultimately activating pro-apoptotic and pro-fibrotic signaling cascades that culminate in systolic and diastolic dysfunction [101]. Similarly, inhibiting DRP1 activity through mitochondrial division inhibitor 1 or enhancing mitofusins-mediated fusion through pharmaceutical drugs like LCZ696 or liensinine ameliorates contractile dysfunction in preclinical models of DbCM, underscoring mitochondrial dynamics as a key therapeutic target [102]. For the mitochondria autophagy pathway in T2DM, contemporary overviews highlight that in a mouse model, cardiac ZIP7 is upregulated and suppresses PTEN-induced kinase 1 (PINK1)/Parkin-dependent mitophagy by lowering mitochondrial Zn^2+^, causing mitochondrial hyperpolarization that prevents PINK1 stabilization and Parkin recruitment. Cardiomyocyte-specific ZIP7 knockout restores mitophagy, reduces mitochondrial ROS, and prevents fibrosis and systolic dysfunction, although evidence for mitophagy flux and relevance to human DbCM remains limited [103].

### 6.4. Oxidative Stress

Oxidative stress in DbCM is driven largely by mitochondrial redox imbalance secondary to lipid oversupply, where increased ETC burden promotes electron leak and superoxide formation (predominantly at complexes I and III) [104], affecting cardiac function by damaging DNA and proteins, and stimulating cells to undergo apoptosis [105]. In addition to mitochondrial and peroxisomal ROS, cytosolic xanthine oxidoreductase (XOR) can contribute to superoxide generation in the diabetic heart. Notably, PKCβ2 activation has been shown to promote XOR-dependent superoxide production via a Rho kinase–c-Jun N-terminal kinase signaling axis in coronary microvessels, suggesting potential positive feedback between lipotoxic PKC signaling and non-mitochondrial ROS sources in DbCM [106,107]. The lack of antioxidant defense intensifies the effects by activating matrix metalloproteinases that impact the structural integrity of the extracellular matrix. ROS production also promotes apoptosis. Cesselli et al. [108] demonstrated that increased ROS was associated with high levels of apoptosis, followed by loss of function in paced dog hearts.

In addition to mitochondrial ROS, peroxisomal β-oxidation also generates hydrogen peroxide (H_2_O_2_) as a by-product of FA metabolism. While peroxisomes do not contribute to ATP production [109], they play a minor role in cardiomyocytes by primarily processing very long chain FAs to maintain redox homeostasis [110]. When peroxisomal ROS generation exceeds detoxification capacity, it can cause oxidative damage, perturb thiol redox status, and disrupt signaling pathways implicated in various human diseases [111].

In turn, ROS induce lipid peroxidation of the inner mitochondrial membrane, increasing its permeability, which impairs mitochondrial membrane integrity and causes the release of pro-apoptotic proteins such as cytochrome *c* into the cytosol, thereby triggering apoptosis and exacerbating myocardial injury [112,113]. In parallel, ROS can oxidize the Fe-S cluster in mitochondrial enzymes such as aconitase, which catalyzes the conversion of citrate to isocitrate in TCA, leading to enzyme inactivation and diminishing the supply of NADH_2_ and FADH_2_ from the TCA cycle [114]. ROS-induced injury of mitochondrial DNA (mtDNA) that encodes genes for OXPHOS is also commonly considered to be a frequent target of mitochondrial ROS [115,116]. In addition, released mtDNA acts as damage-associated molecular patterns, initiating innate immunity by activating pathways like cyclic GMP-AMP synthase-stimulator of interferon genes (cGAS-STING), Toll-like receptor 2/4, and inflammasomes when it escapes the mitochondria into the cytosol or extracellular space, leading to activation of type I interferon responses and inflammatory pathways, respectively, thereby exacerbating cardiac dysfunction.

### 6.5. Treatment of HF by Targeting Lipid Abnormalities

Despite major advances in glucose-lowering and CVD therapies, no treatment is currently approved specifically for DbCM, and clinical diagnosis remains largely based on exclusion and non-specific imaging findings. Metformin remains the primary treatment option for T2DM [117] and can prevent glucose and lipid metabolism dysfunction by activating AMPK, therefore mitigating cardiomyocyte apoptosis, fibrosis, and cardiac insufficiency [37]. Glucagon-like peptide 1 and sodium-glucose cotransporter 2 inhibitors also contribute to promising therapy of HF [118], with the former increasing myocardial glycose oxidation rates and causing weight loss in patients [119]; and the latter can enhance myocardial metabolism, restore mitochondrial function, and prevent programmed cell death [120].

In preclinical models, CD36-null mice were protected against high-fat-diet-induced cardiac steatosis and functional decline, an effect associated with reduced myocardial FA uptake and lipid accumulation [121]. Consistently, in ob/ob mice, CD36 deficiency alleviated cardiac steatosis while attenuating NADPH oxidase-dependent ROS production. Isolated cardiomyocyte studies further demonstrated that loss of CD36 reduces palmitate-induced ROS generation, thereby normalizing redox homeostasis [122]. Similarly, GPAT-deficient mice exposed to high-sucrose or high-fat diets exhibit markedly reduced myocardial TG accumulation compared to wild-type controls, suggesting that limiting upstream lipid synthesis confers cardioprotection under metabolic stress [123]. Pharmacologic zDHHC blockade (e.g., 2-bromopalmitate/CMA) or FoxO1 inhibition/knockdown de-acylates CD36, normalizes substrate handling, and rescues cardiac function [124].

The transcription factor family of PPARs also acts as an effective target of treatments. Whereas PPARα activation has been associated in some models with excessive lipid uptake and ectopic storage, PPARβ/δ promotes oxidative metabolism while maintaining lipid homeostasis [125]. Furthermore, PPARβ/δ confers antioxidant protection by upregulating catalase gene expression, which mitigates oxidative stress-induced apoptosis in cardiomyocytes [126]. Thiazolidinediones and fibrates are types of drugs targeting PPARγ and PPARα, respectively [127]. For instance, thiazolidinediones promote PPARγ activation, which exerts protective effects by lowering FA and TG levels in circulation and enhancing glucose oxidation levels in rat hearts [128]. Similarly, fibrates activate PPARα, which also reduces FA oxidation and circulating free FA levels in the liver and kidney [129]. In obese/diabetic rodent hearts, genetic or pharmacological inhibition of either S6K1 or SREBP1c prevents the onset of lipotoxicity and effectively preserves contractile performance. These findings demonstrate the anti-lipotoxic strategy for treating DbCM [59].

MCD knockout mice hearts displayed significantly higher glucose oxidation levels and less lipid intermediates compared to wild-type mice, following a low-fat diet for 12 weeks [130]. In addition, MCD inhibition increases malonyl-CoA, thereby suppressing CPT1-mediated mitochondrial FA entry and shifting substrate utilization toward glucose oxidation, which has been reported to alleviate lipotoxic stress in preclinical diabetic models [95,96]. On the other hand, in a mouse model of HFpEF, stimulating mitochondrial FAO through cardiac ACC2 deletion improved mitophagy and preserved cardiac energetics, leading to better cardiac function despite increased FAO flux [131]. Moreover, the mitochondria-targeted tetrapeptide elamipretide (SS-31) that concentrates on the inner mitochondrial membrane and binds to cardiolipin preserves cristae and thereby improves mitochondrial OXPHOS while limiting electron leak and ROS. Recent work suggests that SS-31 is associated with restoration of mitochondrial glutathione and increased mitoGPX4 mRNA/protein expression, consistent with reduced lipid peroxidation [132]. However, as GPX4 enzymatic activity was not directly assessed, and evidence for dose-dependent regulation of GPX4 expression is limited, the extent to which SS-31 augments GPX4 anti-ferroptotic function remains uncertain [132].

A common treatment for HF is implantation of a left ventricular assist device, which compensates for defects due to HF, leading to a reduction in mechanical load on the cardiac tissue. This intervention both lowered myocardial ceramide levels and improved whole-body and cardiac insulin sensitivity [133]. In diabetic mouse models, enhancing PGC1α reduces ceramide amount in the myocardium and prevents DbCM [47]. In addition, preserved ER function prevents excessive DAG and TG accumulation in mouse HFpEF, which is essential to protect cardiac function in diabetes [51].

## 7. Future Perspectives

Future work in DbCM should move beyond cataloguing lipotoxic pathways toward defining causal, phenotype-specific mechanisms in humans. Although preclinical studies implicate dysregulated lipid uptake/partitioning and organelle stress as central drivers of DbCM, robust links between specific lipid species and clinical HF phenotypes across the diabetic spectrum remain incompletely established. In particular, the contribution of distinct subcellular lipid pools (e.g., ER-, mitochondrial-, and lipid droplet-associated compartments) to these phenotype-specific trajectories is still poorly defined. Recent mechanistic advances nonetheless point to convergent bottlenecks: maladaptive SREBP-driven lipid influx/lipogenesis [60], impaired XBP1s–ER proteostasis that disrupts lipid-droplet turnover and amplifies oxidative/inflammatory signaling [54], and emerging regulators such as p17/PERMIT [93] and ZIP7–Zn^2+^ signaling [103] that reprogram mitochondrial quality control, collectively coupling lipid overload to redox-inflammatory amplification and energetic failure. Translational progress will therefore require stronger human in vivo causal validation and therapeutic strategies that target these convergent nodes to improve outcomes beyond glycemic control.

## 8. Conclusions

DbCM represents a major and under-recognized contributor to HF in diabetes, and mounting evidence supports cardiac lipotoxicity as a central disease driver rather than a secondary epiphenomenon. Chronic nutrient surplus and insulin resistance impair the balance of myocardial lipid delivery and uptake, utilization, and storage, overwhelming metabolic flexibility, promoting the accumulation of toxic lipid intermediates, and being associated with disrupted mitochondrial function. Consequently, impaired lipid homeostasis induces lipotoxicity in cardiomyocytes, leading to oxidative stress, cell death, and/or myocardial inflammation, leading to HF. Despite substantial mechanistic advances, much of the evidence remains derived from animal or preclinical systems, and causal validation in human DbCM is still limited, which constrains translational confidence and therapeutic development. Strengthening human-relevant models and establishing causal links between lipid dysregulation and clinical phenotypes will be essential to enable effective therapies that improve outcomes beyond glycemic control.

## Data Availability

No new data were created or analyzed in this study. Data sharing is not applicable to this article.

## Abbreviations

The following abbreviations are used in this manuscript:

AMPK	AMP-activated protein kinase
ATGL	adipose triglyceride lipase
CD36	cluster of differentiation 36
CoA	coenzyme A
CPT1/2	carnitine palmitoyltransferase 1/2
CVD	cardiovascular disease(s)
DAG	Diacylglycerol
DbCM	diabetic cardiomyopathy
DGAT	diacylglycerol acyltransferase
DRP1	dynamin-related protein 1
ER	endoplasmic reticulum
ETC	electron transport chain
FA/FAs	fatty acid(s)
FABP3/4	fatty acid-binding protein ¾
FAO	fatty acid oxidation
GPAT	glycerol-3-phosphate acyltransferase
HF	heart failure
HFpEF	heart failure with preserved ejection fraction
HFrEF	heart failure with reduced ejection fraction
HSL	hormone-sensitive lipase
LD	lipid droplet(s)
LDL	low-density lipoprotein
LV	left ventricle
LVEF	left ventricular ejection fraction
MCD	malonyl-CoA decarboxylase
OXPHOS	oxidative phosphorylation
PAK3	p21-activated kinase 3
PINK1	PTEN-induced kinase 1
PKC	protein kinase C
PLN	phospholamban
PPARs	peroxisome proliferator-activated receptors
ROS	reactive oxygen species
SREBPs	sterol regulatory element-binding proteins
SS-31	mitochondria-targeted tetrapeptide elamipretide
STING	stimulator of interferon genes
T2DM	type 2 diabetes mellitus
TCA	tricarboxylic acid cycle
TG/TGs	triacylglyceride(s)
XBP1s	spliced X-box binding protein 1
